# Progressive Hypoxia Exacerbates Breast Cancer Metastasis by Paracrine Modulation of the Blood-brain Barrier Endothelium

**DOI:** 10.1007/s12031-026-02537-6

**Published:** 2026-05-12

**Authors:** Kyle Alan Brinders, David Fisher, Chontrelle Willemse, Khayelihle Brian Makhathini

**Affiliations:** 1 Department of Medical Biosciences, Faculty of Natural Sciences, University of the Western Cape, Bellville, Cape Town, 7535 South Africa; 2 School of Health Professions, University of Missouri, Columbia, MO USA

**Keywords:** Blood-brain barrier, circulating tumour cells, paracrine factors, normoxic, physioxic, hypoxic

## Abstract

Cancer remains a major global concern, with metastasis responsible for most cancer-related deaths. Disseminated cancer cells become circulating tumour cells (CTCs) that travel until arresting at preferential vascular sites such as the blood-brain barrier (BBB), where they cross into the central nervous system (CNS). Given that the BBB robustly maintains CNS homeostasis, the mechanism whereby CTCs metastatically enter the brain is largely unknown. The triple-negative breast cancer (TNBC) (MDA-MB-231 cell line) cells were cultured under normoxic (21% O_2_), physioxic (5% O_2_), and hypoxic (1.5-2% O_2_) conditions to generate conditioned media (CM). Diluted CM was used to treat brain endothelial cells (BECs: bEnd.5 cell line). Endothelial suppression of cell division was assessed using the trypan blue exclusion assay. Morphological changes and an abnormality index were evaluated using hematoxylin and eosin (H&E) staining. Transendothelial electrical resistance (TEER) measured the BEC monolayer integrity following CM exposure. Comparatively, all oxygen-derived CM suppressed BEC suppression of cellular division; however, hypoxic-derived CM was most severe, reducing cell division by 67.7% at 96 h. H&E staining similarly showed that hypoxic-derived CM induced an abnormality rate of 980 abnormalities per 1 000 cells. TEER measurements demonstrated that the hypoxic-derived CM overwhelmed BEC homeostatic mechanisms, significantly increasing monolayer permeability. A clear pattern of progressive dysfunction correlated with decreasing oxygen tension in MDA-MB-231 cell cultures. While the normoxic and physioxic CM had comparable effects, hypoxic-derived CM elicited exacerbated responses, with substantial impairment detectable as soon as 24 h. These findings suggest hypoxic tumour secretomes overwhelm the endothelial regulatory mechanisms, leading to BEC dysfunction and, ultimately, BBB compromise.

**Graphical Abstract:**

**Figure:**
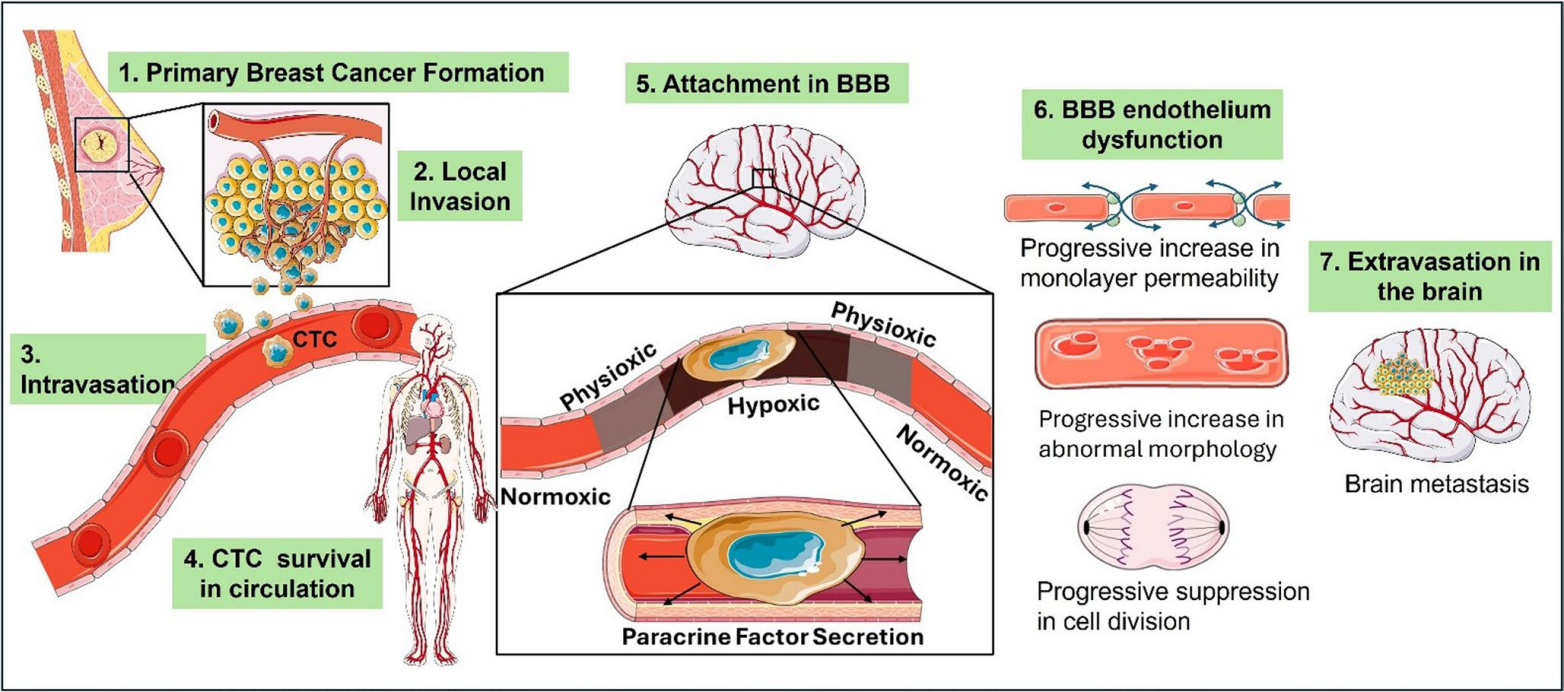
Illustration of breast cancer circulating tumour cells (CTCs) origin and metastasis to the blood-brain barrier (BBB). Illustrations from Server Medical Art (https://smart.servier.com), used under CC BY 4.0 license (https://creativecommons.org/licenses/by.4.0/).

**Supplementary Information:**

The online version contains supplementary material available at 10.1007/s12031-026-02537-6.

## Introduction

In cancer patients, metastasis accounts for 90% of the cancer-associated mortality (Li et al. 2025, Liu et al. 2021). In 2022, a worldwide estimate of 19.3 million new cancer cases and approximately 10 million cancer deaths were recorded. Breast cancer is the leading cause of cancer-related deaths in women worldwide (Bray et al. 2024). Metastases are the successive processes that take place in which cancer cells from the primary tumour dissociate and spread to a distal, secondary location, forming a secondary tumour (Follain et al. 2020, Park et al. 2022). The risk of brain metastasis increases up to 46% for patients with triple-negative breast cancer (TNBC), with metastatic breast cancer cells possessing organotropism to the brain (Witzel et al. 2016, Rostami et al. 2016, Lin 2013).

For brain metastasis to occur, cancer cells must cross the normally impermeable blood-brain barrier (BBB). The BBB is a highly regulated membrane that plays a crucial role in maintaining the delicate environment of the central nervous system (CNS) by performing a critical role in the maintenance of homeostatic regulation thereof by excluding blood-borne toxins, chemicals, hormones, and pathogens that may be in the peripheral circulatory system from the brain *milieu* (Atluri et al. 2015, Kadry et al. 2020, Alahmari 2021, Mentor and Fisher 2022). Although the functional unit of the BBB is composed of pericytes and astrocytes, the specialized brain endothelial cells are the primary regulatory component and express tight junction proteins, decreasing paracellular permeability and creating a capillary barrier lacking fenestrations (Tunon-Ortiz and Lambid 2019, Stamp et al. 2023). A disruption of the BBB structure, or of its primary component, the capillary endothelium, leads to severe compromise of the homeostatic regulation of the brain’s neuronal environment (Chen et al. 2022, Che et al. 2024). During the process of metastasis, circulating tumour cells (CTCs) have the ability to breach the robust BBB. However, there is a scarcity of literature reporting the mechanism by which these CTCs (primary tumour cells that have undergone adaptation and intravasation into the circulatory system) traverse the BBB.

The rationale for this study is the effect of a luminally attached CTC on the brain microvasculature, which can be categorised into direct and indirect effects on the vessel. The indirect effects occur as these CTCs circulate in the bloodstream during the metastatic process until they reach a preferred distal capillary bed, where they attach to the apical plasma membrane of the capillary endothelium. Given the narrowness of the capillary lumen, which is designed to allow only single-file passing of erythrocytes, the attached CTC, effectively blocks capillary blood flow, reducing oxygen supply (Perea Paizal et al. 2021). The attachment of CTCs to the apical surface of the capillary lumen leads to the formation of local hypoxic zones in the blood vessel. The severity of the impairment of blood flow in the capillary can be explained by the law of Poiseuille, in which there is a direct correlation between the diameter of the blood vessel and the blood flow rate by a fourth power (Humayun et al. 2021, Ritchie et al. 2021). The effect of these zones of hypoxia on the ability of the CTC to affect the compromise of the brain capillary endothelium is unknown. Furthermore, the direct effect of CTCs occurs as a result of the CTC secretome, as CTCs have been shown to promote extravasation through their secretome (Humayun et al. 2021, Ritchie et al. 2021). It is therefore important to investigate the role of the secretome in promoting the CTC transition across the capillary endothelium into the brain.

This study aimed to determine and compare the direct effects of TNBC (MDA-MB-231 cell line) cultured under decreasing oxygen tensions [normoxic (21% O_2_), physioxic (5% O_2_), hypoxic (1.5-2% O_2_)] and to expose their harvested paracrine factors (CTC secretome) to brain endothelial cells (bEnd.5 cell line). In clinical breast cancer, cells typically undergo a 2-to-7-day period during which they must survive, adapt, and ultimately extravasate across the vascular wall, processes that are essential for the establishment of brain metastases (Wrobel and Toborek 2016). The rationale for the 96-hour exposure was to model sustained exposure of brain endothelial cells to tumour-derived paracrine factors under various oxygen tensions, rather than a single transient ischemic episode – simulating an attached CTC in the cerebral microvasculature. This study emphasizes the metastatic relevance of the CTC secretome and how oxygen deprivation progressively increases CTC secretion of paracrine factors, which, independent of other factors, affects the pathophysiology of brain capillary endothelial cells.

## Methods and Materials

### Experimental Design

**Fig. 1:**
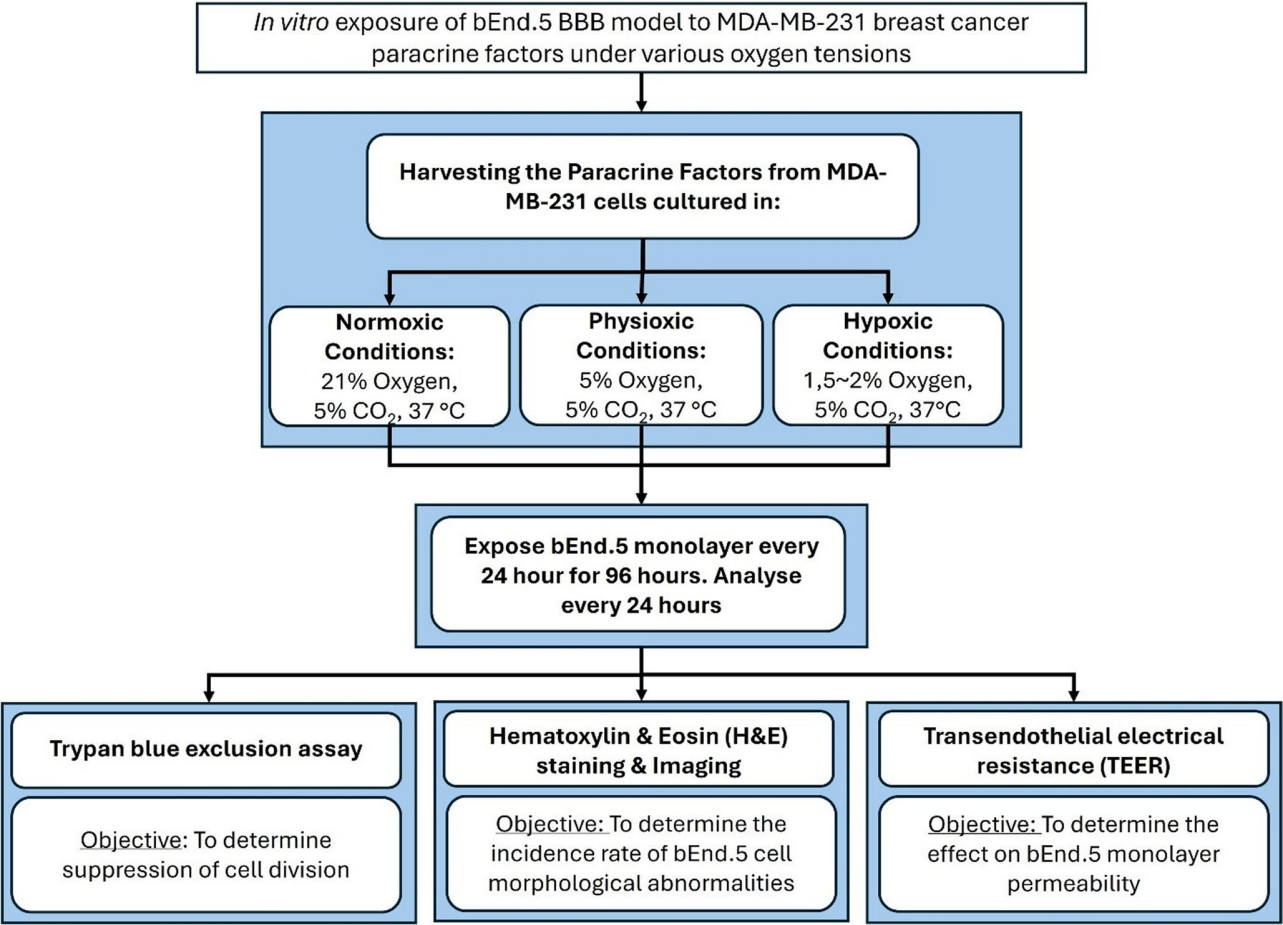
Flow chart displaying the experimental design to investigate the effect of triple-negative metastatic breast cancer (MDA-MB-231) paracrine factors at various oxygen tensions on the structure and function of the brain endothelial cell (BEC) line, bEnd.5 monolayer

The outline of the experimental design is schematically represented in Fig. 1. This study investigated the effect of triple-negative breast cancer (TNBC) (MDA-MB-231) paracrine factors on the brain endothelium cells (bEnd.5 cell line). In this study, paracrine factors refer to the supernatant collected from the MDA-MB-231 cells, where conditioned media (CM) refers to the dilution of the specified concentrations of the paracrine factors. Thus, CM will be used as opposed to concentrations of paracrine factors within this article. In preparation for this experimental approach, the paracrine factors needed to be harvested from the MDA-MB-231 cell line, conditioned, and prepared at various oxygen tensioned environments as per (Rado et al. 2021). The oxygen tensions used within this study were normoxic (21% O_2_), physioxic (5% O_2_), and hypoxic (1.5-2% O_2_). The rationale for treating the bEnd.5 cells at various oxygen tensions was for the increasing relevance of the progressive decrease in oxygen availability, as a result of the CTC, in which hypoxic conditions become relevant (Rado et al. 2021, Bertout et al. 2011).

The media was replaced daily for all exposure groups and controls to maintain cellular function and the substrates of bEnd.5 cells. The bEnd.5 cells were exposed to the CM, and after every 24 h for 96 h, cell division, viability, and toxicity were evaluated using the trypan blue exclusion assay. Subsequently, Hematoxylin & Eosin (H&E) staining was performed to determine the incidence of morphological abnormalities. In addition, transendothelial electrical resistance (TEER) was measured to assess the effect on monolayer permeability. All experiments were carried out in triplicate (*n* = 3) and duplicated for repeatability.

### Cell Lines and Bio-Reagents

This experiment used the bEnd.5 cell line (ECACC, Sigma-Aldrich, 96091930, St. Louis, MO, USA) representing the brain endothelial cells of the blood-brain barrier, and the MDA-MB-231 cell line (RRID: CVCL_0062) (donation from Dr. B Abrahams, Faculty of Medicine and Health at the University of Free State) a triple negative breast cancer (TNBC) cell line representing the metastatic cancer. The bEnd.5 cells and normoxic (21% O_2_) cultured TNBC cells were cultured and maintained in a physiologically replicative environment of 37 °C in an incubator with 5% humidified CO_2_. This environment was standard for the bEnd.5 cell line; however, the MDA-MB-231 cell line was subjected to various experimental environmental conditions (physioxic [5% O_2_] and hypoxic [1.5-2% O_2_]; Fig. 1).

The culture media Dulbecco’s Modified Eagle Medium: Nutrient mixture F-12 (DMEM/F12, Gibco, Thermofisher, Cat no, 11320074) was supplemented with 10% Fetal Bovine Serum (FBS) (Gibco Gamma irradiated, Thermofisher, Cat no, 10493-106), 1% Penicillin Streptomycin (Pen-Strep) (Gibco, Thermofisher; Cat no, 15140-122), 1% Sodium Pyruvate (NaP) (Gibco, Thermofisher, Cat no, 11360-039) and 1% Non-Essential amino acids (MEM NEAA) (Gibco, Thermofisher, Cat no, 11140-050). This media composition was used as the base media for all cell cultures in this study. It was also used to dilute the harvested TNBC supernatant when preparing the conditioned media (CM). Analytical grade 0.25% trypsin-EDTA (Gibco, Thermofisher Cat no, 25200-072), phosphate-buffered saline (PBS) (Whitehead Scientific, BE17-517Q), and 0.4% trypan blue (Gibco; Cat no, 15250061) were used throughout the research study.

### Harvesting of the Paracrine factors: Conditioned Media

The MDA-MB-231 cells were seeded at a density of 5 × 10^6^ cells/T_175_ flask, at a final volume of 50 ml, and allowed for growth and adherence for 24 h at 37 °C in an incubator with 5% CO_2_ and 95% relative humidity. This seeding process was implemented across all three environments (normoxic, physioxic, and hypoxic). After 24 h of cellular adherence, the spent media were replaced with 50 ml of fresh, supplemented media, and the T_175_ flasks were prepared for harvesting paracrine secretion under each environmental condition. For the collection of normoxic (21% O_2_) paracrine factors, the flasks were incubated at 37 °C, 5% CO_2_, and 95% relative humidity for 48 h. For both the physioxic (5% O_2_) and hypoxic (1.5–2% O_2_) environments, flasks were placed into a sterilised modular incubator hypoxia chamber (MIC 101; Billups-Rothenberg, Inc., Sorrento Valley Blvd, San Diego, CA 92121, USA), and the chamber was placed in an incubator at 37 °C for 48 h. A Greisinger oxygen meter with a sensor (GOX 100-0-CO. No 600437, Billups-Rothenberg, Inc., San Diego, CA, USA) was used to measure oxygen levels and to obtain the physoxic (5% O_2_) and the hypoxic (1.5-2% O_2_) environments. Following the 48 h, the paracrine factors (supernatants) were collected and stored on ice, centrifuged at 2 500 rpm, and stored at -80 °C until further use. The MDA-MB-231 CM were prepared at concentrations of 20%, 40%, and 75% by diluting the thawed supernatant with supplemented DMEM/F12 to attain the desired concentrations.

### Trypan Blue Exclusion Assay

To investigate the effect of the paracrine secretion on the cellular division of the bEnd.5 cells, the trypan blue exclusion assay was performed. This assay uses trypan blue to indicate cells with a compromised cellular membrane: cells that dye blue following exposure are categorised as dead, whereas those that do not are categorised as live. These categorizations are further used for downstream calculations (Kamiloglu et al. 2020).

The bEnd.5 cells were seeded into a 12-well plate at 2.5 × 10^3^ cells/ml/well, in triplicate, and the cells were incubated at 37 °C in an incubator at 5% CO_2_ and at 95% relative humidity for 24 h to allow for attachment. Thereafter, the bEnd.5 cells were exposed to various concentrations of CM for 96 h. Plate termination was done every 24 h using 0.25% Trypsin-EDTA. The cells were centrifuged at 2 500 rpm for 5 min, and the supernatant was discarded. The cell pellet was then resuspended in 400 µl of fresh media. To perform the trypan blue exclusion assay, 20 µl of the cell suspension was mixed with 20 µl of 0.4% trypan blue, in a 1:1 ratio. 10 µl of the mixture was loaded onto the dual-chamber hemocytometer. Cells were counted using the Countess™ 3FL Automated Cell Counter (2188A21010554, Invitrogen, Thermo Fisher Scientific). The total, live, and dead cells were recorded simultaneously by the Countess. The total, live, and dead cells were used for the downstream calculations of suppression of cellular division. Cellular division suppression was calculated by subtracting the total cell number in the exposure group from that in the control group (Fig. 3). This calculation provided the level of suppression within each exposure group.

### Hematoxylin and Eosin (H&E)

bEnd.5 cells were seeded onto Poly-L-Lysine (Cat no. P8920, Sigma-Aldrich, Merck, South Africa) coated coverslips (22 × 22 mm) at a density of 8 × 10^3^ cells/ml/well within a 6-well plate. The cells were allowed 24 h for growth and attachment, whereafter the cells were exposed to their respective oxygen-tensioned conditioned media (CM) as indicated within Fig. 1. At termination, every 24 h until 96 h, the following process was implemented; The coverslips were fixed in 10% buffered formalin (Cat no. FOR001; Kimix Chemical and Lab supplies, Cape Town, South Africa), dehydrated using a series of ethanol at 70, 95, and 100% for 5 min each prior to staining, stained with Hematoxylin Mayers stain (Cat no. HMY2.5; Kimix Chemical and Lab supplies, Cape Town, South Africa), rinsed in running distilled water, stained in Eosin stain solution 1% (Cat no. EOY001; Kimix Chemical and Lab supplies, Cape Town, South Africa) and again rinsed in running water, followed by dehydrating steps and a clearing in Xylenes (Cat no. 33817–2.5 L; Sigma-Aldrich, Cape Town, South Africa), where after the coverslips were mounted using DPX mounting medium (Cat no. 06522-100mL; Sigma-Aldrich, Merck, South Africa).

An abnormality index analysis was conducted. Abnormalities in the control group and all exposure groups were recorded per 1 000 cells per concentration, imaged, and statistically analyzed. Abnormalities included binucleation, multinucleation, abnormal division, irregular nuclear shape, and cytoplasmic blebbing. The distinguishing observations used for these classifications were compared with those of the control group. Bi/multinucleated cells were those observed to possess > 1 nucleus per cell. Irregular cell division included cells displaying incomplete or abnormal cytokinesis. Irregularly shaped nuclei were observed with areas greater than those of control cells, and the nuclei were visibly distorted and non-oval. Cytoplasmic blebbing was classified as cells with spherical membrane protrusions. The abnormality index was determined by quantifying cells exhibiting these predefined morphological abnormalities. Each cell was counted only once; if a single cell displayed multiple abnormality classifications, it was recorded as a single abnormal cell and not counted multiple times.

### Transendothelial Electrical Resistance (TEER)

TEER was used to evaluate the permeability and integrity of the bEnd.5 cell monolayer. The TEER readings were taken using the Millicell epithelial Volt-Ohm meter (Millipore, Ser. No. 57318, 11B, Germany). A chopstick-style electrode was placed in the apical and basolateral chambers, respectively, and an alternating current (AC) voltage was applied to the monolayer, with the resulting current being measured. The Volt-Ohm meter used the principle of Ohm’s law (R = V/I) to monitor the resistance across endothelial layers (Kamiloglu et al. 2020, Srinivasan et al. 2015).

Membrane inserts (HA-Millipore, Cat no. PIHA01250, Germany) with a diameter of 12 mm and a pore size of 0.45 μm were placed into wells of a 12-well plate containing 600µL (basolateral side representation) of culture media, and the inserts were filled with 400µL (apical side representation) culture media. The plates containing the inserts were then incubated for 24 h to allow the inserts to acclimate. The bEnd.5 cells were seeded onto the acclimated inserts at 5 × 10^4^ cells/400µL/insert. The cells were incubated at 37 °C, 5% CO_2_, and 95% relative humidity. The cells were allowed to attach for 24 h. At 48 h, the TEER readings commenced; the 12-well plate was removed from the incubator and placed in a laminar flow hood for an acclimation period of 30 min.

TEER across the bEnd.5 monolayer was conducted with the purpose of investigating the effect of metastatic breast cancer (MDA-MB-231) paracrine factors harvested under normoxic (21% O_2_), physioxic (5% O_2_), and hypoxic (1.5–2% O_2_) conditions on the permeability of the confluent monolayer. The monolayer was exposed to the CM every 24 h for an experimental period of 96 h (Fig. 1). However, it is important to note that the exposure to the CM only began once the monolayer had been established, represented by a plateau in the monolayer’s resistance reading, which occurred at Day 5.

The TEER calculation was performed by subtracting the Blank (the inserts without cells) reading from the experimental reading obtained, and that value was multiplied by the surface area of the inserts, providing the TEER value, as derived from (Rado et al. 2022).

### Statistical Analysis

Statistical analysis was performed using GraphPad Prism software (version 9.5.1, GraphPad Software, San Diego, CA; RRID: SCR_002798). Data were expressed as mean ± SEM. The differences between groups were analyzed using one-way ANOVA with Bonferroni post hoc tests for parametric data, and nonparametric analysis was performed using Dunn’s test. Statistical significance was accepted at *p* < 0.05 for a 95% confidence interval.

## Results

### The Effect of Oxygen Tension Derived Conditioned Media (CM) on BEC Suppression of Cellular Division

The effect of MDA-MB231 normoxic (21% O₂), physioxic (5% O₂), and hypoxic (1.5–2% O₂) fractionalised paracrine factors on bEnd.5 suppression of cellular division was examined by counting the number of live cells every 24 h for 96 h following CM exposure at 20%, 40%, and 75% of paracrine secretion.

**Fig. 2:**
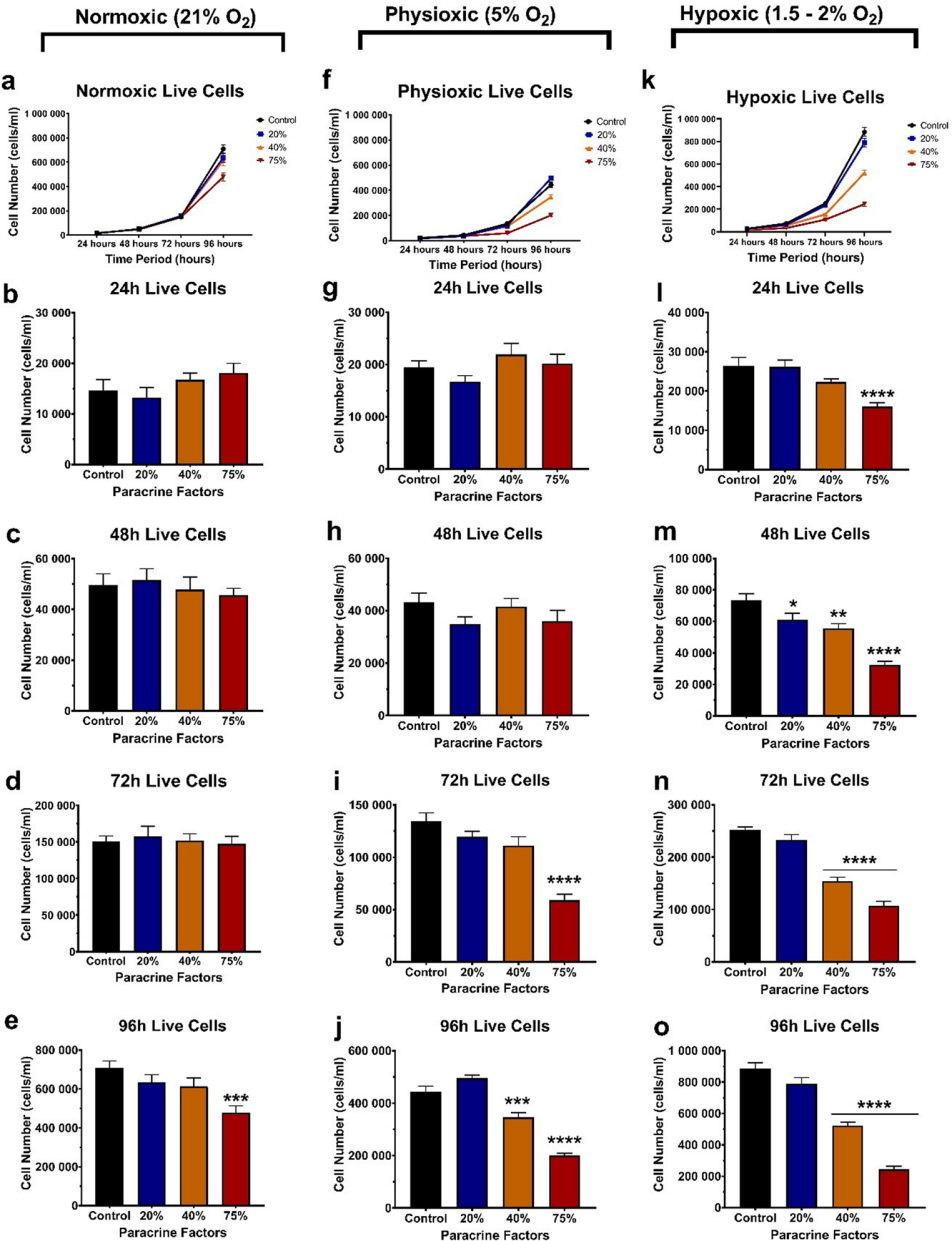
The effect of normoxic (21% O₂), physioxic (5% O₂), and hypoxic (1.5–2% O₂) paracrine factors on bEnd.5 cellular division. **a** Normoxic live cell number following CM exposure over 96 h. **b-e** bEnd.5 daily suppression of cellular division over 96 h following exposure to normoxic-derived paracrine factors. **f** Physioxic live cell number following CM exposure over 96 h. **g-j** bEnd.5 suppression of cellular division over 96 h following exposure to physioxic-derived paracrine factors. **k** Hypoxic live cell number following CM exposure over 96 h. **l-o** Illustrate the bEnd.5 cell suppression of cellular division over 96 h after exposure to hypoxic-derived paracrine factors. Statistical significance was assessed with one-way ANOVA. Data are presented as mean ± SEM (*n* = 6). *p < 0,05, **p < 0,01, ***p < 0,001, ****p < 0,0001

**Fig. 3:**
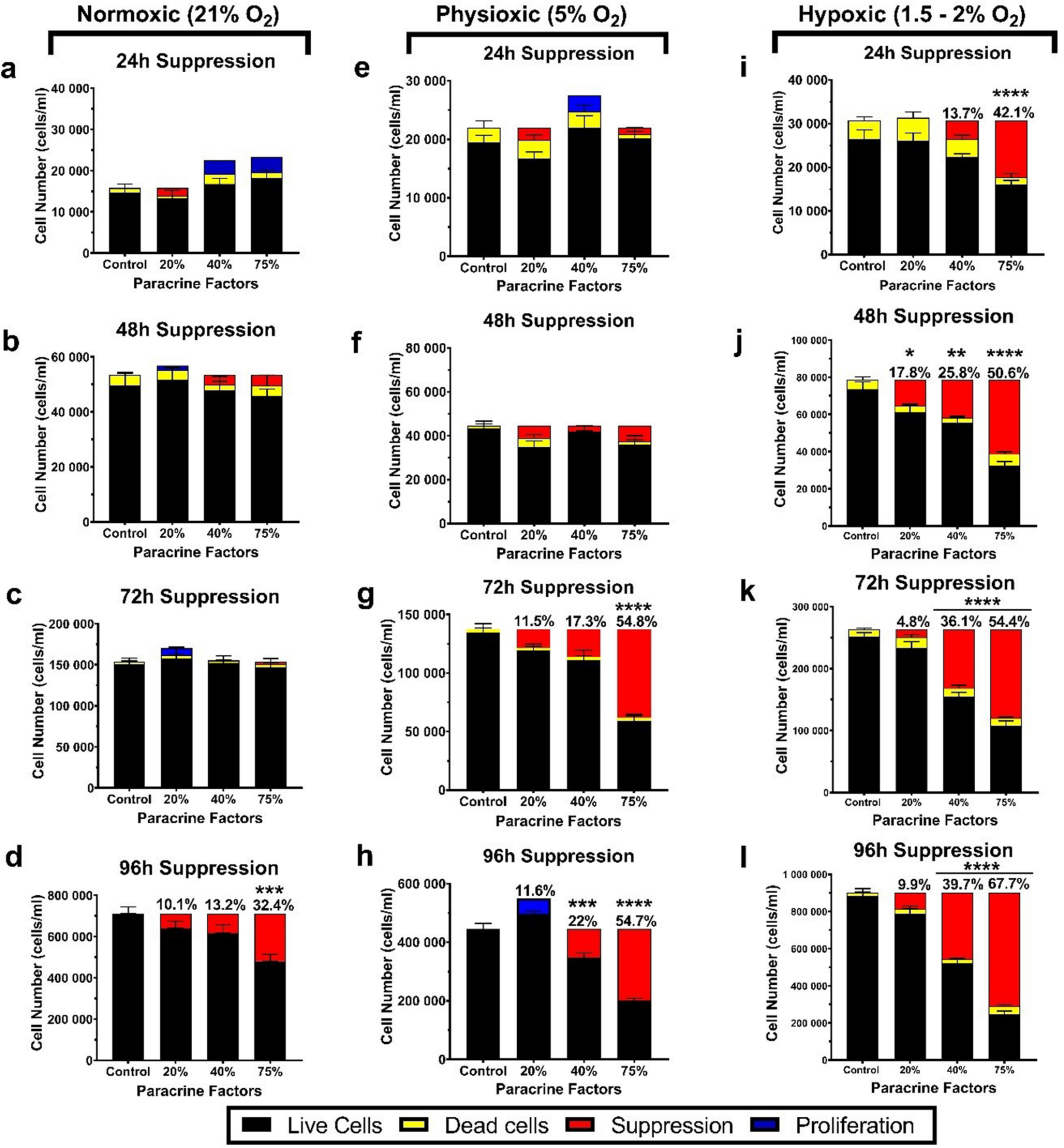
The effect of normoxic (21% O₂), physioxic (5% O₂), and hypoxic (1.5–2% O₂) paracrine factors on bEnd.5 suppression of cell division compared to the control group. **a-d** bEnd.5 cell division suppression over 96 h following exposure to normoxic-derived paracrine factors. Graphs (**e-h**) illustrate bEnd.5 cell division suppression over 96 h after exposure to physioxic-derived secretions. Graphs (**i-l**) show the suppression of bEnd.5 cell division over 96 h after exposure to hypoxic-derived paracrine factors. Statistical significance was assessed using one-way ANOVA. Data are presented as mean and SEM (*n* = 6). *p < 0,05, **p < 0,01, ***p < 0,001, ****p < 0,0001

Under normoxic (21% O₂) conditions, no significant difference in suppression of cellular division was observed across all concentrations from 24 to 72 h compared to the control (Fig. 2a-e), whereas at 96 h, 75% CM showed a significant decrease relative to the control group (Fig. 2e). This decrease in live cell numbers was not due to an increase in dead cells, but was primarily due to 32.4% suppression of cell division compared to the control, as presented at 96 h in the 75% exposure group (Fig. 3d).

In the bEnd.5 experimental group that was treated with physioxic CM (5% O_2_; Fig. 2f-j), no significant effects were observed over 48 h across the different concentrations of the CM (Fig. 2g-h). Treatment with 75% CM produced a significant decrease in the suppression of cellular division at 72 h (Fig. 2i). The effects at 96 h are demonstrated in Fig. 2j, where both the 40% and 75% CM caused significant reductions in the suppression of cellular division.

In this group of experiments, where bEnd.5 cells were treated with physioxic CM, suppression of cell division was observed at the 72-hour time point, with a significant reduction in the 75% exposure group, corresponding to a 54.8% decrease in cell number (Fig. 3g). Additionally, at 96 h, cell suppression was observed with decreases of 22% and 54.7% for the 40% CM and 75% CM, respectively (Fig. 3h).

Exposure of bEnd.5 cells to a hypoxic CM (1.5–2% O₂) demonstrated a significant dose-dependent effect across the exposure times (Fig. 2k-o). The 40% CM and 75% CM groups showed the most notable decrease in suppression of cellular division compared to the control group (Fig. 2l-o).

The decrease in live cell numbers in these experiments also resulted in consistent suppression of cell division in both 40% and 75% CM-treated groups (Fig. 3i-l), ranging from 13.7% to 39.7% for 40% CM and 42.1% to 67.7% for 75% CM. Statistical resolution for the suppression of cell division occurred only at 48 h, despite the tendency observed at 72 and 96 h.

**Fig. 4:**
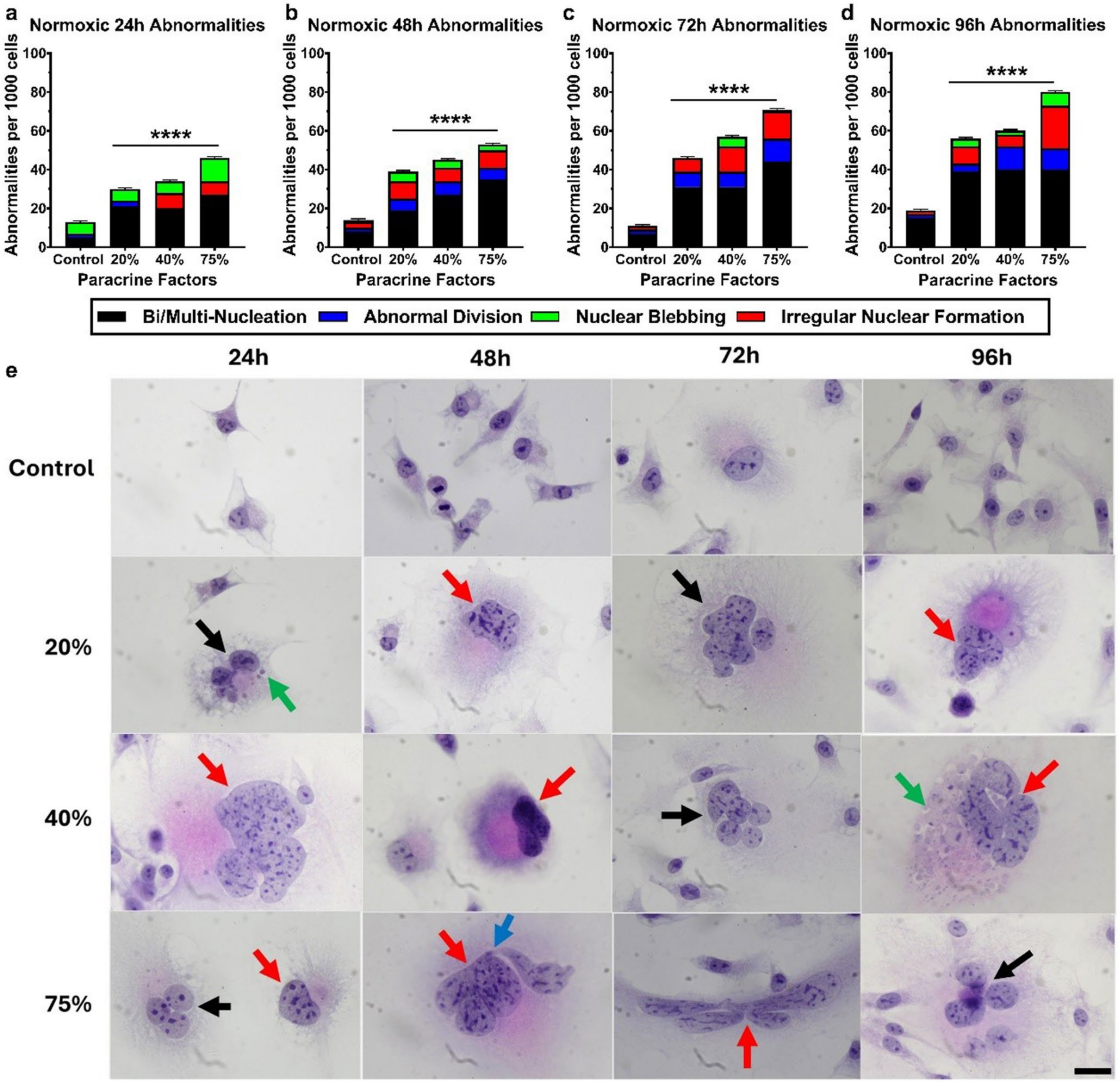
The effect of normoxic (21% O_2_) MDA-MB-231 paracrine factors on the morphology of bEnd.5 cells. The bEnd.5 cells were treated with varying concentrations (20%, 40%, and 75%) of paracrine factors and stained with H&E at 24, 48, 72, and 96 h post-exposure. Black arrows indicate bi- and multi-nucleated cells; red arrows indicate irregular nuclear formation; green arrows indicate nuclear blebbing; blue arrows indicate abnormal cellular division. Images were captured at 100X magnification. The scale bar at the bottom right displays 20 μm for all images. Statistical significance was determined with one-way ANOVA. Data are presented as mean ± SEM (*n* = 6). ****p < 0,0001

The effect of normoxic CM on the structure of exposed bEnd.5 cells was assessed every 24 h over a 96-hour period using H&E staining. Figure 4 offers a graphical summary of the number of abnormalities per 1,000 cells at each time point, compared to the control, along with annotated histological images highlighting these abnormalities. A significant increase in abnormalities was observed across all concentrations during the 96 h. The most common abnormalities identified during this period were bi- and multi-nucleation (black arrows), as well as irregular nuclear formation (red arrows). Nuclear blebbing (green arrows) was noted at 20% after 24 h and at 40% after 96 h; additionally, abnormal cellular division (blue arrows) was evident at 75% after 48 h. The histological images of the control and CM exposure groups after 96 h show noticeable changes in nuclear morphology and an increase in cell size.

**Fig. 5:**
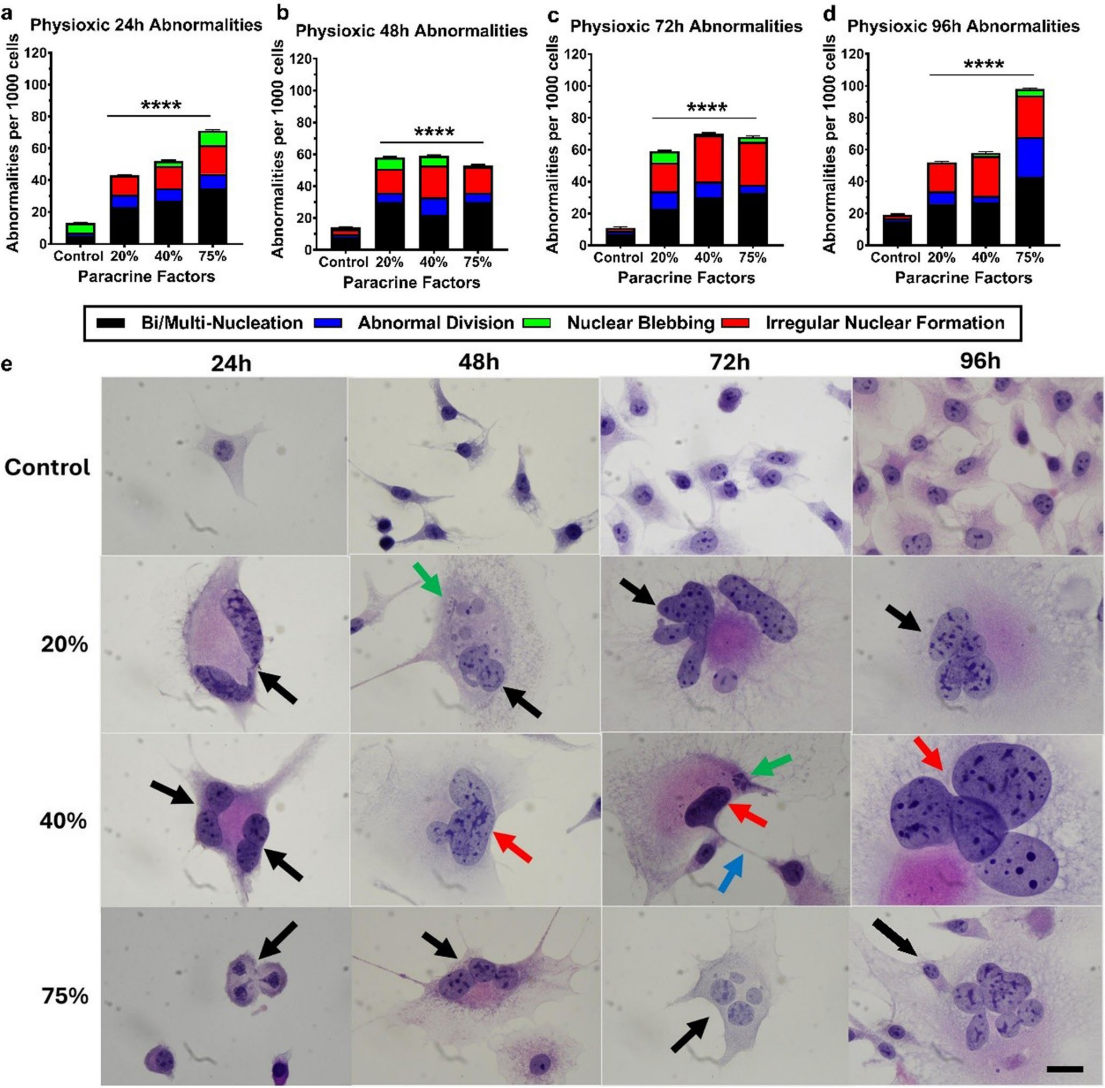
The effect of physioxic (5% O2) MDA-MB-231 paracrine factors on the morphology of bEnd.5 cells. bEnd.5 cells were treated with various concentrations (20%, 40%, and 75%) of paracrine factors and stained with H&E at 24, 48, 72, and 96 h after exposure. Black arrows indicate bi- and multi-nucleated cells; red arrows indicate irregular nuclear formation; green arrows indicate nuclear blebbing; blue arrows indicate abnormal cellular division. Images were captured at 100X magnification. The scale at the bottom right shows 20 μm for all images. Statistical significance was determined with one-way ANOVA. Data are presented as mean ± SEM (*n* = 6). ****p < 0,0001

The effect of the physioxic CM on bEnd.5 cell structure every 24 h over 96 h was assessed using H&E staining. Figure 5 compares the number of morphological abnormalities per 1,000 cells in each sample group versus the control at each time point, showing that exposure groups have a statistically significant increase in abnormalities at all time points. The most common abnormality observed was bi- or multinucleation (black arrows) of the brain endothelial cells, which occurred at all time points across all exposure groups. At 48 h, nuclear blebbing (green arrows) was noted, along with abnormal cellular division (blue arrows) at 72 h in the 40% exposure group. The 96-hour group exhibited more irregular nuclear formations (red arrows) than at earlier time points. Histological images of the controls and the CM-exposed groups at 96 h showed effects on nuclear morphology and an increase in cell size compared with the control group.

**Fig. 6:**
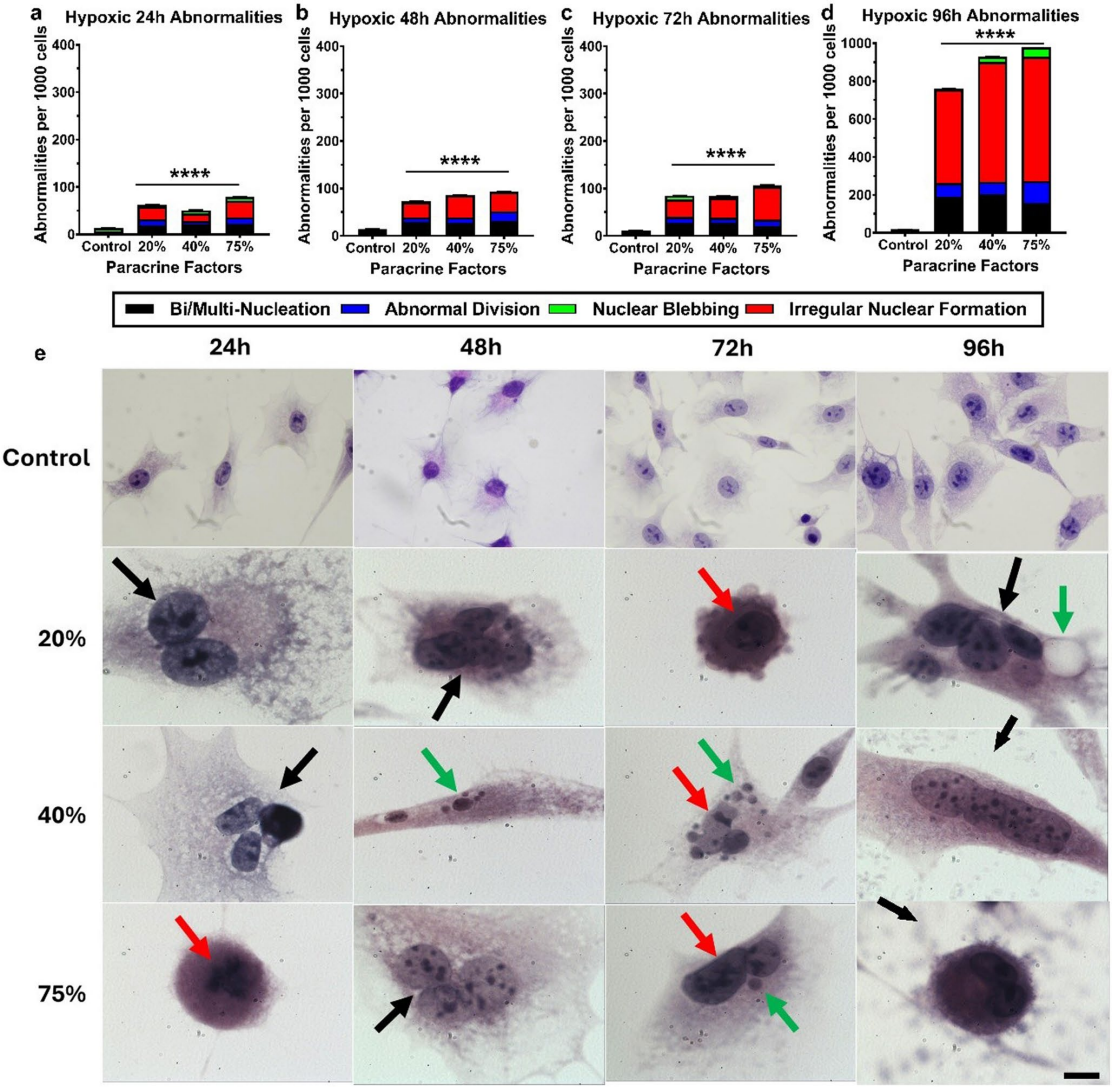
The effect of hypoxic (1.5-2% O_2_) MDA-MB-231 paracrine factors on the morphology of bEnd.5 cells. bEnd.5 cells were treated with various concentrations (20%, 40%, and 75%) of paracrine factors and stained with H&E at 24, 48, 72, and 96 h after exposure. Black arrows indicate binucleated and multinucleated cells; red arrows indicate irregular nuclear formations; green arrows indicate nuclear blebbing. Images were taken at 100X magnification. The scale bar at the bottom right represents 20 μm for all images. Statistical significance was determined with one-way ANOVA. Data are presented as mean ± SEM (n = 6). ****p < 0,0001

The effect of hypoxic CM on the structure of exposed bEnd.5 cells was assessed every 24 h using H&E staining. Figure 6 compares the number of morphological abnormalities per 1,000 cells in each sample group relative to the control at each time point, showing that hypoxic CM caused a statistically significant increase in abnormalities at all times. Cellular analysis indicated that all time points exhibited a significant rise in abnormalities compared to the control, with nearly a tenfold increase between the 72- and 96-hour marks. Throughout the 96-hour experiment, the hypoxic CM exposure groups showed extensive bi-nucleation, multi-nucleation (black arrows), and nuclear blebbing (green arrows). The 72-hour period revealed a high occurrence of irregular nuclear formations (red arrows). Histological images of the controls and the CM exposure groups at 96 h demonstrated clear effects on nuclear morphology and an increase in cell size compared to the control group.

### The Effect of Oxygen Tension Derived Metastatic Breast Cancer Paracrine Factors on BEC Monolayer Permeability

TEER was used to assess the functionality of the brain capillary endothelial monolayers (bEnd.5 cell) by measuring their permeability after exposure to normoxic (21% O_2_), physioxic (5% O_2_), and hypoxic (1.5-2% O_2_) paracrine factors. TEER readings were taken daily from days 1 to 5 to verify the formation of a confluent and physiologically functional monolayer, as shown by the plateauing of TEER values.

**Fig. 7:**
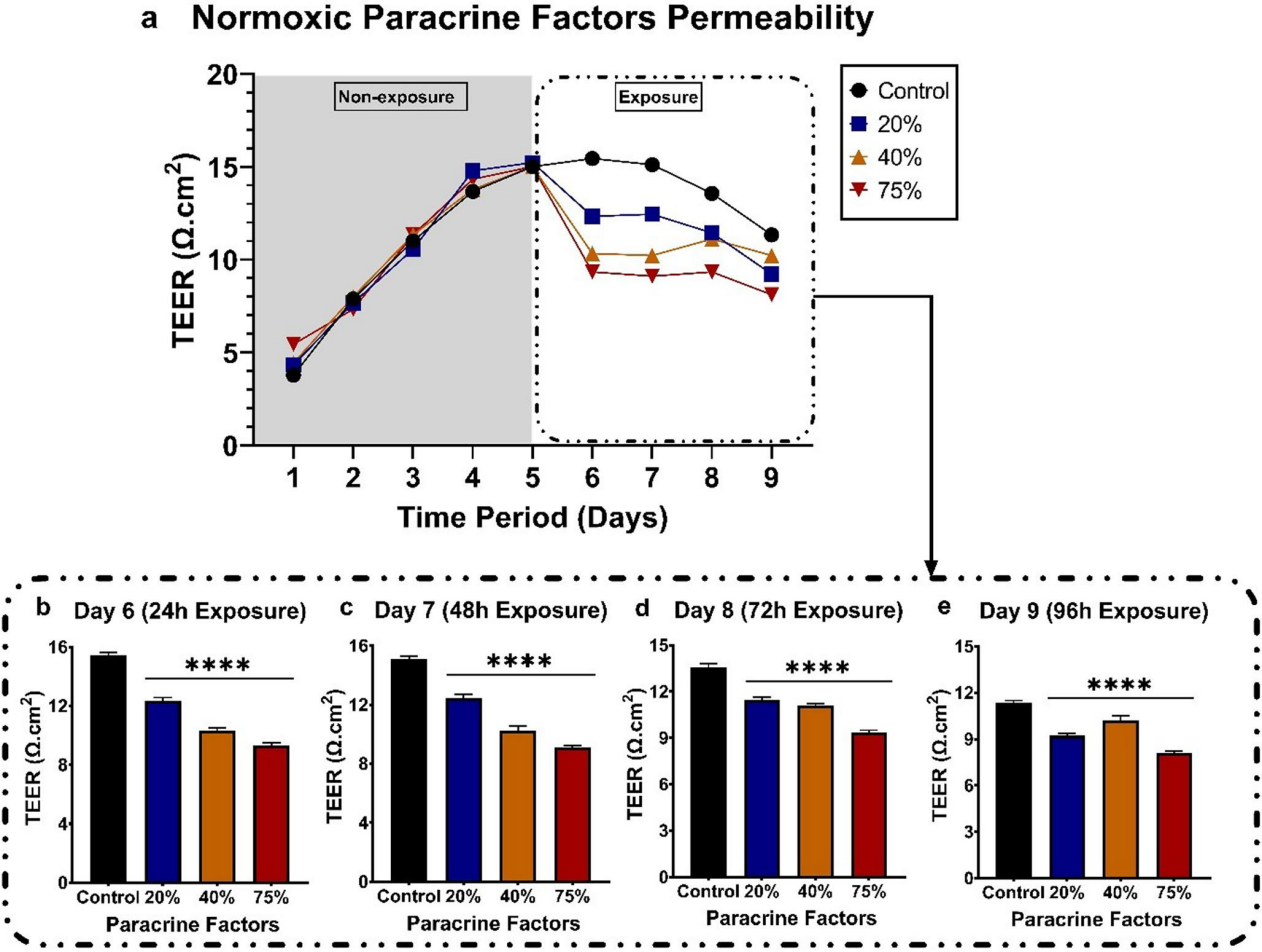
The effect of MDA-MB-231 paracrine factors grown under normoxic (21% O_2_) conditions and exposed to the bEnd.5 cell monolayer. a) Line graph showing the formation of the monolayer before exposure and during the 4-day (96-hour) exposure period. b-e) The impact of normoxic paracrine factors on the bEnd.5 monolayer at various concentrations (20, 40, and 75%), measured every 24 h over 96 h. Statistical significance was determined using one-way ANOVA. Data are presented as mean ± SEM (*n* = 6). **p < 0,01, ***p < 0,001, ****p < 0,0001

After a confluent monolayer formation on day 5, the monolayer was exposed to normoxic (21% O_2_) paracrine factors at 20%, 40%, and 75% CM. Figure 7b-e emphasise the effect of the normoxic (21% O_2_) paracrine factors shown in Fig. 7a. Figure 7b-d display a dose-dependent decrease in TEER readings observed over the 72-hour exposure period. Although Fig. 7e does not follow this trend, all exposure groups still exhibited a significant increase in monolayer permeability.

**Fig. 8:**
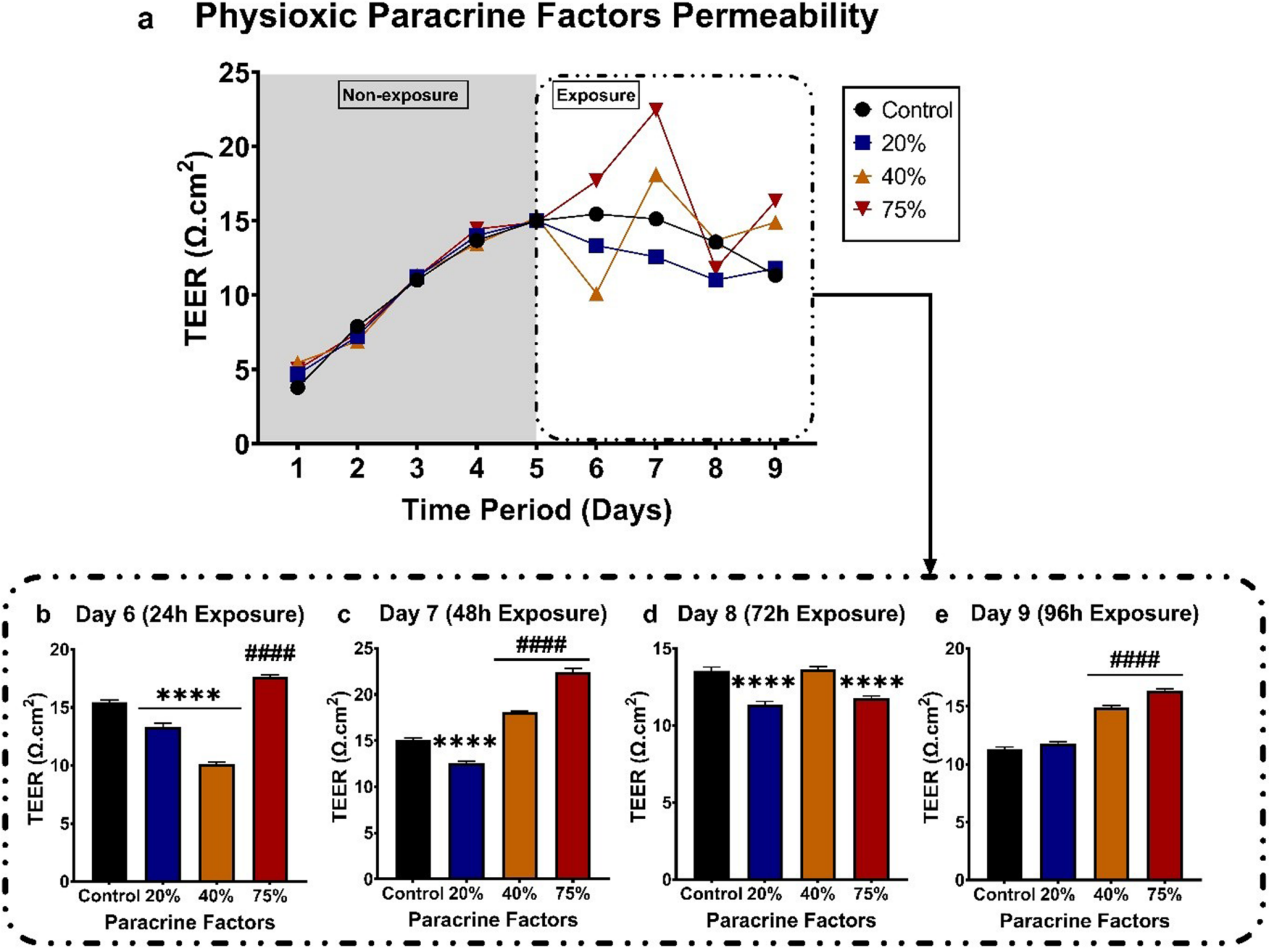
The effect of late-stage breast cancer (MDA-MB-231) paracrine factors grown in physioxic (5% O2) conditions at 20%, 40%, and 75% on the bEnd.5 cell monolayer. **a** Line graph showing the establishment of the monolayer before exposure and during the 4-day (96-hour) exposure period. **b-e** The effect of physioxic paracrine factors on the bEnd.5 monolayer at various concentrations every 24 h over 96 h. Statistical significance was determined with one-way ANOVA. Data is shown as mean ± SEM (*n* = 6). *Indicates a decrease with significance. ****p < 0,0001. # Indicates an increase with significance. ####*p* < 0.0001

After formation, the monolayer was exposed to physioxic (5% O_2_) paracrine factors at 20%, 40%, and 75%. Figure 8b-e display the statistical significance of daily exposure to the CM throughout the experiment. In Fig. 8a, a pattern emerged where, at higher CM concentrations, TEER oscillated with increases and decreases over the exposure period. Figure 8b demonstrated a significant decrease in TEER after 24 h of exposure (day 6) for the 20% and 40% CM exposure groups, and an increase in TEER was observed at the 75% CM. Figure 8c showed a significant decrease in the 20% CM exposure group, and an increase in TEER for the 40% and 75% CM exposure groups after 48 h of exposure (day 7). Figure 8d illustrates the monolayer’s response to the physioxic CM after 72 h of exposure, indicating a statistically significant decrease in TEER for the 20% and 75% CM exposure groups. A reversal of the trend seen at 72 h is observed at 96 h (Fig. 8e), where an increase in TEER in the 40% CM and 75% CM exposure groups was observed.

**Fig. 9:**
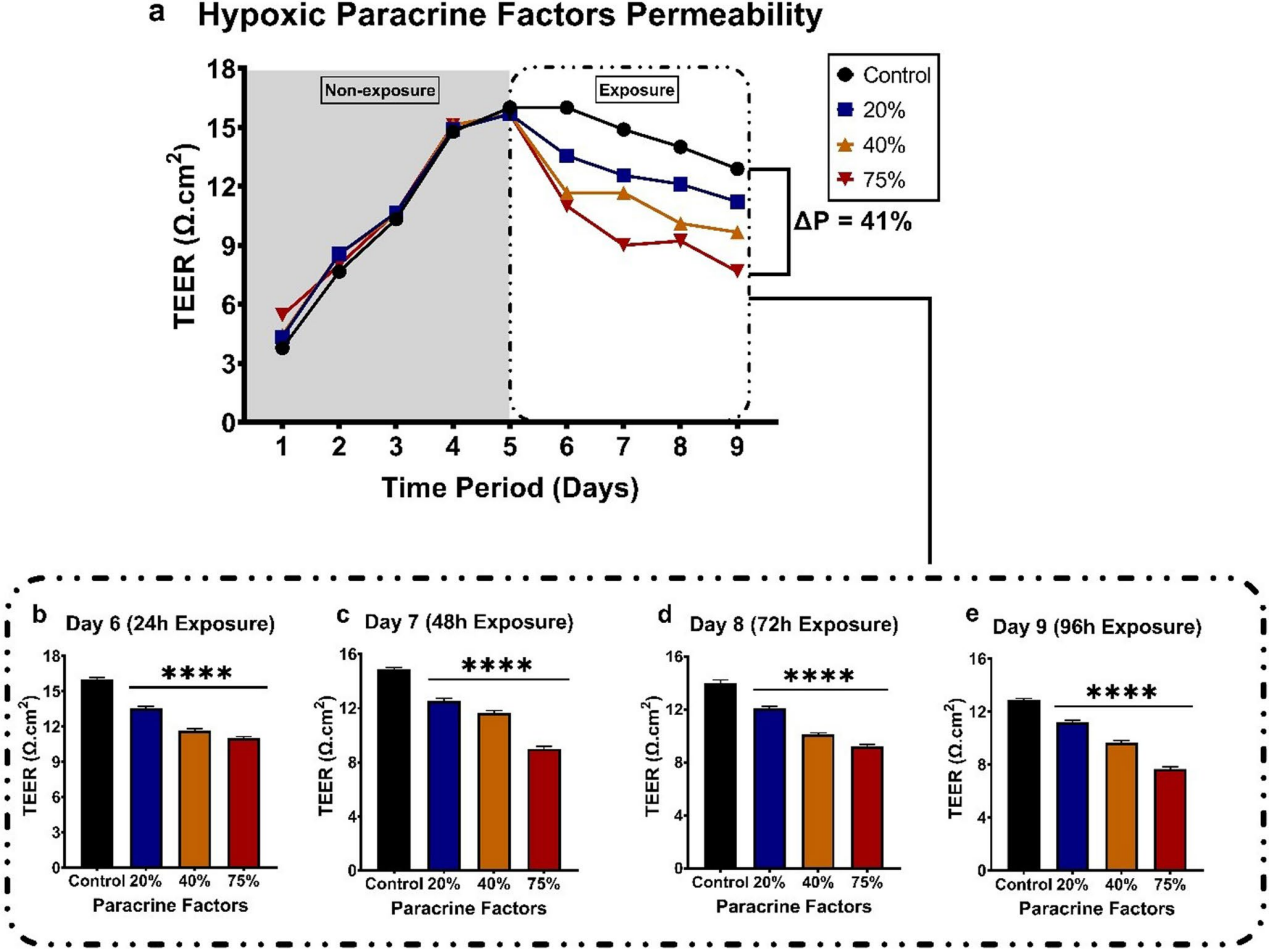
The effect of late-stage breast cancer (MDA-MB-231) paracrine factors grown in hypoxic (1.5-2% O_2_) conditions at 20%, 40%, and 75% on the bEnd.5 cell monolayer. **a** The monolayer was established (Days 1–5) before exposure to paracrine factors (Days 5–9). ΔP represents a decrease by 41% in monolayer TEER of the 75% CM group compared to the control. **b-e** The monolayer was exposed every 24 h for 96 h. Statistical significance was determined with one-way ANOVA. Data are presented as mean ± SEM (*n* = 6). **p < 0,01, ***p < 0,001, ****p < 0,0001

The effect of hypoxic (1.5-2% O_2_) CM on the bEnd.5 monolayer is illustrated in Fig. 9, with Fig. 9a providing a linear overview of the 9-day experimental period. The percentage difference between the control and TEER for the 20%, 40%, and 75% CM exposure groups after 96 h is 13.8%, 26%, and 41%, respectively (ΔP in Fig. 9a ). Figure 9b-e demonstrates a consistent dose-related decrease in TEER readings and shows a substantial reduction in TEER for all exposure groups across the 96-hour exposure period.

## Discussion

Cancer incidence is increasing globally and is rising with population growth, with more than a 70% increase in cases estimated by the year 2050 (World Health Organization 2024). In 2022, a global estimate of 19.3 million new cancer cases and 10 million cancer deaths was recorded, with breast cancer being the leading cause of cancer-related deaths in women worldwide, followed by lung and colorectal cancer (Bray et al. 2024). The risk of developing brain metastasis as a secondary site ranges up to 30%, with the brain being the most affected organ in approximately 12% of breast cancer patients; however, this risk increases with TNBC to 46% (Rostami et al. 2016, Lin 2013). Breast cancer metastatic cells have a preference to attach to luminal apical plasma membranes of brain capillaries (BBB), dramatically impairing blood flow in the affected capillary, thereby decreasing blood perfusion to both the capillary endothelium and the affected capillary bed, decreasing oxygen, and leading to reduced oxygen tension (Perea Paizal et al. 2021, Rado et al. 2022). The decreased perfusion across the capillary facilitates the local accumulation of paracrine factors in the blood capillary. Poiseuille’s law, which states that the flow rate through a vessel is proportional to its radius to the fourth power (Yamaki et al. 2021, Cao et al. 2019), illustrates the drastic effects of CTCs on impeding blood flow. This process indirectly exacerbates pathological outcomes, as impaired perfusion leads to hypoxia-induced endothelial injury and paracellular barrier breakdown, ultimately resulting in functional disruption of the BBB (Wrobel and Toborek 2016, Sharma et al. 2025, Rado and Fisher 2022). We investigated whether CTCs also “directly” affect the brain capillary endothelium via their paracrine factors.

While the use of one cancer cell line was executed in this study, we do not consider this a limitation. Many cancers metastasize to distant organs, the mechanisms enabling brain access differ between cancer types. Metastasis is therefore not a uniform process; different cancers exhibit distinct metastatic behaviours and organotropism, resulting in preferential colonisation of specific secondary sites rather than random dissemination (McDonald et al. 2023, Wang and Luo 2021). This study therefore focused on the paracrine tumour-endothelial interactions of the TNBC, as even within a single cancer type, subtypes can employ different mechanisms for brain metastasis and BBB transversal (Dai et al. 2025). While this approach aligns with established in vitro models of tumour-endothelial signaling, it limits generalisations across cancer subtypes and endothelial phenotypes. Nonetheless, the use of a single cancer cell line remains valuble for mechanistic investigation of TNBC metastasis.

In this study, the simulation of selected hypoxic zones of the luminally attached CTC in the microvasculature of the brain was conducted by implementing selected ambient oxygen environments: normoxic (21% O_2_), physioxic (5% O_2_), and hypoxic (1.5-2% O_2_) for cultured metastatic breast cancer cells. We hypothesized that reduced oxygen tension, which occurs in vivo when metastatic breast cancer cells attach to the apical surface of brain capillaries and impair local perfusion, induces focal capillary hypoxia, prompting tumour cells to secrete elevated levels of paracrine factors. These factors, in turn, may independently compromise the physiological and morphological integrity of the brain capillary endothelium. Therefore, this research examined the effect of MDA-MB-231 cell-derived CM from different oxygen tensions on bEnd.5 suppression of cellular division, cellular morphology, and BEC monolayer permeability over a 96-hour exposure period. It is important to note that attempting to understand the direct effects of the MDA-MB-231 cancer cell secretosome on the BECs, the bEnd.5 cells were always cultured under normoxic conditions, thus controlling for bEnd.5 changes in oxygen tension.

### The Effect of Oxygen Tension-derived Conditioned Media on BEC Suppression of Cellular Division

The specialized endothelial cells are a critical element of the BBB and play a core role in the interactions between the peripheral circulatory system and the CNS (Li et al. 2025). However, when breast cancer cells metastasize and arrest in the cerebral microvasculature, they incur a direct effect on the endothelial cells, ultimately causing structural and functional alterations to the BBB (McDonald et al. 2023). In this study, we utilised the metastatic MDA-MB-231 cancer cell line cultured under normoxic (21% O_2_), physioxic (5% O_2_), and severe hypoxic (1.5-2% O_2_) conditions. We compared the rate of bEnd.5 cell division over 96 h across all oxygen-tensioned CM (Fig. 2a, f and k). Our initial observation was that bEnd.5 cells did not statistically respond to either normoxic or physioxic conditioned media at 24 to 48 h (Fig. 2); however, at 96 h, a significant decrease was seen in both normoxic and physioxic treatments with the 75% conditioned media (Fig. 2e and j, respectively). These data indicated that BECs were surprisingly resilient to submaximal concentrations of paracrine factors; however, higher concentrations affected their morphology and suppressed cell division. Secondly, treatment with paracrine factors derived from hypoxic conditions showed a dose-dependent effect on bEnd.5 cells from as early as 24 h to 96 h, where 40% CM caused suppressed cell division of 13.7% to 39.7% and 75% CM suppressed cell division by 42.1% to 67.7% (Fig. 3).

A study by (Rado and Fisher 2022), showed that non-metastatic breast cancer cells (MCF7) caused a significant reduction in cell viability after 96 h of exposure to bEnd.3 cells cocultured with MCF7 cells. They also demonstrated that the effects of MCF7 conditioned media produced under normoxic and physioxic conditions exhibited a statistical effect on their bEnd.3 cellular division at 72 and 96 h (Rado et al. 2021). Additionally, they observed that exposure to physioxic MCF7 conditioned media supernatant had a more pronounced effect on bEnd.3 cells than when exposed to normoxic MCF7 conditioned media. Based on our current findings, which show the impact of the CM derived from culturing MDA-MB-231 cancer cells under different oxygen tensions, on treating the bEnd.5 cells, aligns with previous research on how breast cancer influences endothelial cell division (Galloni et al. 2024).

### The Effect of Oxygen Tension-derived Conditioned Media on BEC Morphology

The results of the suppression of cellular division study led to the investigation of the effects of the paracrine factors on the structure and morphology of the bEnd.5 cells.

Morphological studies have shown that morphological abnormality is often associated with functional compromise. Thus, it was of interest to investigate whether treatment with CM derived from the MDA-MB-231 cancer cell line would compromise the morphology of brain capillary endothelial cells. Confluent endothelial monolayers exhibit reduced cell division due to contact inhibition, limiting opportunities to observe abnormal mitosis. To address this, we compared morphological irregularities between cultures exposed to CM and an untreated control, and given that we observed suppression in the rate of cellular division, we wanted to investigate whether this was due to morphological pathology induced by the CM.

The abnormality counts of the normoxic (Fig. 4a-d) and physioxic-derived (Fig. 5a-d) paracrine secretion exposed bEnd.5 cells exhibited a similar incidence of morphological abnormality throughout the 96-hour experimental period, with the final rates of their abnormalities resulting in 80 and 100 abnormalities per 1,000 cells, respectively. CM generated under normoxic and physioxic conditions produced comparable, moderate levels of morphological disruption (Figs. 4 and 5), suggesting that the endothelial cells can partially compensate for the associated paracrine stress. In contrast, the hypoxic-derived CM elicited a pronounced, dysregulated spectrum of abnormalities, all hallmarks of unresolved mitotic stress as early as 24 h (Fig. 6a). This observed severity was aggravated over the remainder of the experiment, and at the 96-hour analysis, the incidence ranged between 760 and 980 morphological abnormalities per 1,000 cells (Fig. 6d). Nuclear morphology changes, such as nuclear blebbing and abnormal nuclear morphology, have been linked to detachment from the lamina, which can lead to significant nuclear instability, including a repeating cycle of nuclear envelope expansion, lysis, and repair. A significant consequence of nuclear instability is increased cellular senescence (Wang and Luo 2021, Dai et al. 2025). The occurrence of abnormal cell division, bi- and multi-nucleation can be linked to the process of interrupted cytokinesis, in which failures can lead to abnormalities such as tetraploidy (Galloni et al. 2024, Stephens et al. 2019). Additionally, this process involves several key cellular regulators, including RhoA, which drives cytokinetic furrow formation at the midbody of dividing cells. The disruption of RhoA activity or spatial localization impairs furrow formation, ultimately preventing the successful completion of cytokinesis (Normand and King 2010). Finally, this escalation in abnormal cell morphology, reflected in the increased release of hypoxia-induced paracrine factors, collectively overwhelms endothelial homeostatic mechanisms. The resulting mitotic failure, which followed the compromised structural integrity of the individual endothelial cells, was one of the most likely causes leading to suppression of cell division.

### The Effect of Oxygen Tension-derived Conditioned Media on BEC Monolayer Permeability

It was important to investigate how oxygen tension-derived CM contributes to the functional destabilization of the confluent BEC monolayer, thereby promoting an increase in the BBB permeability and supporting conditions for CTC extravasation across the BBB into the CNS.

A key feature of the BBB is the presence of tight junction proteins, which create a tight cell-to-cell structure that forms a non-fenestrated microvasculature in the brain. The high electrical resistance associated with the BBB mainly results from these tight junctions and their restriction of paracellular transport across the barrier (Stamp et al. 2023, Ivey et al. 2009).

CTCs that metastasize to the brain microvasculature exert a significant effects on BBB endothelial cells via indirect mechanisms (induced capillary hypoxia due to impeded blood flow) and direct mechanisms, such as secreted paracrine factors. CTC adhesion to the luminal wall of the endothelium results in paracrine factors, which contain inflammatory cytokines and proteins such as matrix metalloproteinases (MMPs), which facilitate metastasis (Li et al. 2022). This increase in MMPs released into the vasculature, together with a decreasing oxygen availability, contributes to endothelial disruption and destabilization, which increases vascular permeability and a pro-tumorigenic environment that promotes extravasation of the tumour cells into the brain (Dhar et al. 2018).

The reduction in oxygen availability to endothelial cells caused by CTCs adhering to the luminal surface of brain capillaries induces hypoxia within the BBB endothelium (Liu et al. 2012). Under hypoxic conditions, cancer cells may enter a state a preferential reprogrammed metabolism, using anaerobic glycolysis (without the need for O_2_), referred to as the Warburg effect (Barba et al. 2024). Furthermore, under glucose deprivation, cancer cells shift to overexpression of GRP94, which upregulates BCL2 and other anti-apoptotic proteins as a survival mechanism (Wang et al. 2021). Additionally, to overcome reactive oxygen species (ROS)-mediated damage, the cancer cells express the transcription factor LEF1 to enhance glutathione production, to counter the effects of ROS generation (Wang et al. 2021). Taken together, these metastatic cancer cell-associated processes suggest a potential mechanism by which extravasation across the BBB could be facilitated; however, further investigation is still warranted to determine their impact on the integrity of the brain capillary endothelium.

In this study, we used TEER to assess the integrity of confluent bEnd.5 monolayers following exposure to MDA-MB-231 breast cancer cell paracrine factors (CM), cultured under selected oxygen tensions to further test our postulated hypothesis. In our findings, all normoxic and hypoxic-exposed groups showed significant, dose-related increases in monolayer permeability throughout the 96-hour exposure period (Figs. 7 and 9). Treating confluent brain endothelial monolayers with physioxic-derived CM, however, showed a biphasic response. This oscillation is seen as a rise and fall in TEER, alternating every 24 h (Fig. 8b-e). These results are supported by the findings of (Rado et al. 2021), in which they cultured bEnd.3 cells exposed to normoxic and hypoxic-derived CM. Given that they cultured the bEnd.3 cells under physioxic conditions (5% O_2_; referred to as hypoxic in the study) showed that bEnd.3 cell cultures cultured in supplemented media under physioxic conditions showed a rise-and-fall trend in TEER, alternating every 24 h. TEER is a dynamic, real-time measure of endothelial barrier integrity that is known to vary over time due to both biological and technical factors. Temporal fluctuations in TEER have been attributed to dynamic changes in junctional morphology and cell-to-cell contact length during endothelial monolayer maturation, reflecting structural remodelling rather than uniform barrier disruption (Srinivasan et al. 2015, Felix et al. 2021). In the context of this study, the oscillatory TEER pattern observed in Fig. 8 under physioxic-derived CM may result from transient endothelial responses to paracrine cues, combined with ongoing adjustments in junctional organisation and endothelial morphology. Such effects may be further influenced by the physiologically relevant oxygen tensioned paracrine secretion, which is known to modulate endothelial signaling and junctional dynamics differently from normoxic culture conditions (Funamoto et al. 2017). Furthermore, previous studies have shown that TNBC, specifically MDA-MB-231 cells, negatively affects BMECs (Fan and Fu 2016, Hamester et al. 2022, Godinho-Pereira et al. 2021). Hamester et al., 2022 (Barba et al. 2024) the three BC cell lines. Using an electric cell-substrate impedance sensing (ECIS) system to analyse endothelial monolayer resistance (similar to TEER), an increase in permeability within the first 10 min after seeding BC cells at a 1:1 ratio onto the BMEC monolayer was observed. Our findings also suggest that the observed biphasic response may reflect homeostatic mechanisms attempting to counteract the effects of the CM generated under physioxic conditions. Under hypoxic conditions, however, amplified paracrine factor secretion appears to exceed these compensatory mechanisms, resulting in a dose-related increase in monolayer permeability and an inability of endothelial cells to maintain homeostatic mechanisms.

### Limitations of the Study

A limitation of this study is to consider that TNBC is a heterogeneous disease, characterized by differences in genomic, transcriptomic, and immunologic profiles (Derakhshan and Reis-Filho 2022). This heterogeneity means that different TNBC cell lines and subtypes may produce distinct secretomes, the collection of secreted factors (CM), that may influence the tumour microenvironment in unique ways, providing additional mechanisms for metastasis across the BBB. For example, given that MDA-MB-231 belongs to the mesenchymal stem-like subtype, and the cell line HCC1806 belongs to the basal-like subtype 2, it may be expedient to compare the CM effects of these two (or more) TNBC cell lines on BBB endothelia, to strengthen the common mechanisms for brain capillary metastasis.

A constraint of the mouse brain endothelial cell line BBB model is the exclusion of other components of the neurovascular unit, such as astrocytes and pericytes. While this approach is consistent with established in vitro models investigating paracrine tumour-endothelial interactions, it limits the generalizability of the findings across different cancer subtypes and endothelial phenotypes.

Although the extensive use of mouse brain endothelial cells as an established model in the literature (Rado et al. 2022, Rado and Fisher 2022, Ku et al. 2016), in terms of a limitation of this study, we acknowledge that the use of a murine brain endothelial cell line may represent a limitation that may restrict the direct extrapolation of our findings to human pathology. However, (Sun et al. 2022) showed that murine cell line (bEnd3) and pluripotent stem cell-derived human brain endothelial cells (ihBMEC) have similar morphological and physiologically functioning, while their TEER measurement values vary; both these in vitro models are suitable to study the effects of pathogen and drug delivery impact on the endothelial cells (Sun et al. 2022).

Additionally, although TEER measurements provided valuable real-time insights into barrier integrity, we did not complement these data with additional assessments such as tight junction protein localisation or expression. Such analyses would strengthen our mechanistic understanding of the observed TEER changes and provide more direct evidence of junctional disruption or reorganisation.

Finally, our findings represent preliminary evidence of the effects of tumour-derived secretomes on BEC function. While the results suggest potential mechanisms by which TNBC metastatic cancer cells may influence BBB integrity, this study did not involve the separation of protein factors (cytokines, growth factors, etc.) from extracellular vesicle-borne factors. This would require further studies to confirm the relevance of these observations to metastatic extravasation and clinical pathology.

## Conclusion

The effects of normoxic and physioxic paracrine factors are similar, with overall impacts after 96 h being alike; in contrast, hypoxic-derived secretions are significantly more severe and noticeable, occurring earlier and with greater intensity. This confirms our proposed hypothesis and provides valuable insights into the damaging effects of the CTC secretome and the consequences of oxygen deprivation resulting from their blockage of cerebral microvascular perfusion.

Our findings support the hypothesis that progressive oxygen deprivation of the CTC secretome following CTC attachment may generate a paracrine factor gradient that influences endothelial function. Increasing hypoxia may therefore contribute to the potency of paracrine factors that compromise endothelial function, thereby potentially explaining a possible mechanism that may facilitate CTC extravasation into the brain. These factors modify capillary endothelial morphology, leading to the suppression of normal cell division, and their cumulative effect progressively impairs endothelial function, ultimately increasing monolayer permeability by 41%, which may reflect mechanisms that could contribute to CTC traversal of the BBB.

Furthermore, this study emphasises the importance of analysing the direct effects of metastasised CTCs and their secretome, in the absence of endothelial hypoxia, as well as the process that may relate to changes in the BEC integrity, potentially offering insight into potential mechanisms of extravasation into the CNS.

## Supplementary Information

Below is the link to the electronic supplementary material.

Supplementary Material 1

## Data Availability

All experimental data collected are at the University of the Western Cape (UWC) and are available in accordance with UWC data and intellectual property policy guidelines and their associated copyright protections.
