# Supplementary material for: Progressive Hypoxia Exacerbates Breast Cancer Metastasis by Paracrine Modulation of the Blood-brain Barrier Endothelium
Source: J Mol Neurosci. 2026 May 12;76(2):82. doi: 10.1007/s12031-026-02537-6 (PMC13167879; doi:10.1007/s12031-026-02537-6)
Supplement: Supplementary file 1 — Supplementary Material 1 [file 12031_2026_2537_MOESM1_ESM.docx]

**Supplementary Material**

**Table S1.** Representative examples and characterisation of each morphological abnormality. Abnormalities identified by a red outline in the representative images. Notice that cells presenting abnormality were significantly larger than control cells. Scale bar = 10µm.

|  | **Representative Images** | **Pre-defined morphological criteria** | **Physiological Implications** | **References:** |
| --- | --- | --- | --- | --- |
| **Control** | 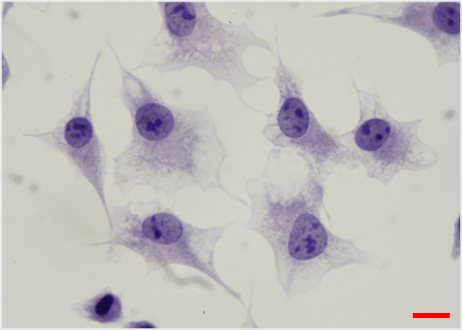 | Spindle-shaped cells with oval-to-round nuclei | N/A | [1], [2], [3], [4] |
| **Bi-/multinucleation** | 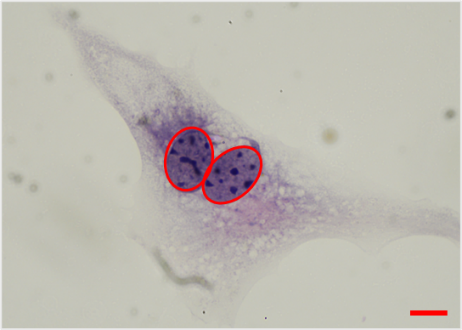 | ≥1 nucleus per cell | Aneuploidy causing mitotic catastrophe |  |
| **Irregular cell division** | 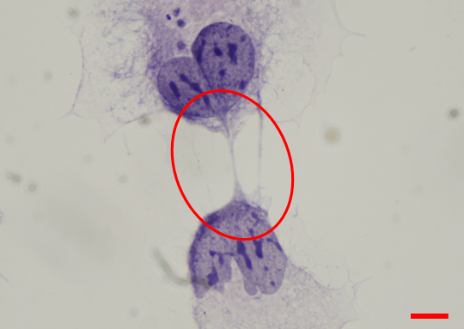 | Incomplete or abnormal cytokinesis | Aneuploidy causing mitotic catastrophe |  |
| **Irregular nuclear shape** | 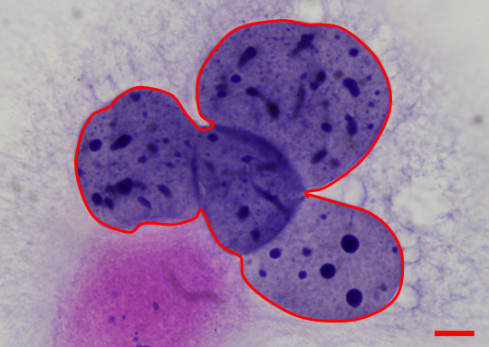 | Nuclear area > control  Visibly distorted or non-oval nuclei morphology | Compromise of cellular function, including cell-cell interactions |  |
| **Cytoplasmic blebbing** | 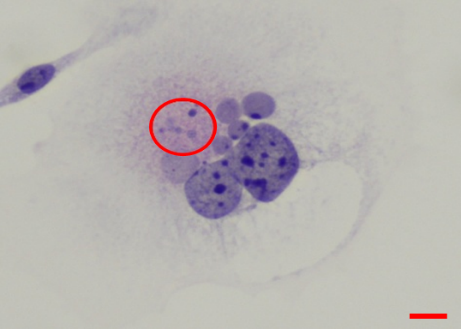 | Membrane protrusions or bulges on the cell surface | Predictive of eventual oncotic cell death |  |

The abnormality index was calculated as the percentage of cells displaying more than 1 abnormal morphology feature per field.

**References:**

[1] D. B. Buglak *et al.*, “Nuclear SUN1 stabilizes endothelial cell junctions via microtubules to regulate blood vessel formation,” *Elife*, vol. 12, Mar. 2023, doi: 10.7554/eLife.83652.

[2] M. Estacion and W. P. Schilling, “Maitotoxin-induced membrane blebbing and cell death in bovine aortic endothelial cells,” *BMC Physiology 2001 1:1*, vol. 1, no. 1, pp. 2-, Feb. 2001, doi: 10.1186/1472-6793-1-2.

[3] N. M. Borradaile and J. G. Pickering, “Polyploidy impairs human aortic endothelial cell function and is prevented by nicotinamide phosphoribosyltransferase,” *Am. J. Physiol. Cell Physiol.*, vol. 298, no. 1, pp. 66–74, Jan. 2010, doi: 10.1152/ajpcell.00357.2009.

[4] E. V. Sazonova, S. V. Petrichuk, G. S. Kopeina, and B. Zhivotovsky, “A link between mitotic defects and mitotic catastrophe: detection and cell fate,” *Biology Direct 2021 16:1*, vol. 16, no. 1, pp. 25-, Dec. 2021, doi: 10.1186/s13062-021-00313-7.
